# Social Determinants of Men’s Health Across the Life Course

**DOI:** 10.1093/geroni/igab046.1629

**Published:** 2021-12-17

**Authors:** Roland Thorpe, Keith Whitfield

## Abstract

There is a paucity of research focusing on the complex interaction between social, behavioral, biological, and psychosocial factors, and health outcomes among men. This symposium contains a collection of papers that discuss some key social determinants of health (SDOH) that can provide insights to advance our understanding of men’s health and aging across the life course. Dawn will discuss the Stroke Counseling for Risk Reduction (SCORRE) intervention designed to increase awareness, risk perceptions, and health behaviors to reduce stroke risk in African Americans. Findings suggest tailoring the intervention to the needs and preferences of young African American men. Archibald and colleagues seek to determine if race differences in allostatic load (AL) among adult men vary by age. Black men 45-64 had a higher AL score (PR = 1.14, 95% CI 1.02, 1.28) than White men. Skipper and colleagues used a grounded theory approach to examine the negative interactions of 35 religious middle and old age Black men. Analyses reveal that church-related negative interactions broadly fall within the following themes: (1) ageism within intergenerational churches, (2) people are messy, and (3) issues with leadership. Bruce and colleagues examine the association between religious service attendance and mortality among Black men. Participants who attended at least once per week were 18% less likely to die than their peers who did not attend a religious service at all (HR 0.82; 95% CI 0.68-0.99). These presentations collectively will bolster our knowledge on key SDOH among men across the life course.

